# Cavernous Sinus Syndrome in a Traumatic Dissecting Aneurysm of the Internal Carotid Artery

**DOI:** 10.7759/cureus.75414

**Published:** 2024-12-09

**Authors:** Chua Ter Wei, Muharliza Musa, Rosilah Mohamad, Safinaz Mohd Khialdin

**Affiliations:** 1 Department of Ophthalmology, Hospital Kuala Lumpur, Kuala Lumpur, MYS; 2 Department of Ophthalmology, Hospital University Kebangsaan Malaysia, Kuala Lumpur, MYS

**Keywords:** cavernous sinus syndrome, exposure keratopathy, fungal corneal ulcer, internal carotid artery aneurysm, traumatic dissecting aneurysm

## Abstract

We report a rare case of a missed intracavernous internal carotid artery dissecting aneurysm occurring as a complication of the base of skull fracture with severe brain injury causing acute cavernous sinus syndrome with permanent vision loss.

A 31-year-old Myanmar lady had an alleged motor vehicle accident and suffered severe traumatic brain injury with multiple intracranial bleeds, multiple facial bone and base of skull fractures, and limb fractures.

At one week post-trauma, she had severe right eye proptosis with vision loss, ophthalmoplegia, chemosis, and high intraocular pressure. She was suspected clinically to have a traumatic cavernous carotid fistula and a lateral canthotomy and cantholysis was performed.

The patient was scheduled for an embolization of the fistula but during cerebral digital subtraction angiography, a dissecting aneurysm instead was noted at the right internal carotid artery at the cavernous segment. A cerebral computed tomography angiography and venography showed right cavernous sinus and right superior ophthalmic vein thrombosis. The patient was started on antiplatelet agents and underwent successful stenting-assisted coiling of the aneurysm, and her right eye proptosis gradually resolved.

While awaiting definitive treatment of the aneurysm, the patient had severe exposure keratopathy and despite intensive hydration and antimicrobial treatment, it later developed into a fungal corneal ulcer. A temporary tarsorrhaphy was done and the ulcer gradually healed into a significant corneal scar and the final visual acuity of her right eye was no light perception.

The underlying cause of cavernous sinus syndrome is diverse and unique. A thorough assessment and appropriate neuroimaging should be performed to arrive at the proper diagnosis in a timely manner. This is in order to prevent delays in the definitive treatment and irreversible ocular morbidity.

## Introduction

Internal carotid artery (ICA) dissections can be caused by trauma or occur spontaneously, where the arterial wall tears and leads to subintimal haematoma causing narrowing of the lumen, occlusion, aneurysmal dilatation, or vascular rupture. ICA dissecting aneurysm (DA) is extremely rare with an incidence of 2.5-3 per 100,000 [1]. This can occur following cervicocranial injury and head and neck surgery [2].

We report a rare case of a missed intracavernous ICA DA occurring as a complication of the base of skull fracture with severe brain injury causing acute cavernous sinus syndrome (CSS) with permanent vision loss.

## Case presentation

A 31-year-old Myanmar lady, who was a pillion rider, had an alleged motor vehicle accident after her motorbike skidded. She sustained polytrauma with severe traumatic brain injury. A plain computed tomography brain scan revealed a base of skull fractures with multiple intracranial haemorrhages and multiple facial bone fractures. Features of cavernous sinus pathology such as asymmetry of the cavernous sinus and dilated superior ophthalmic vein were not seen in the initial scan. She also sustained fractures of the right arm, foot, and pubic rami. She was initially admitted to a private centre and was treated by a multidisciplinary team including neurosurgery, orthopaedics, otorhinolaryngology, and oral & maxillofacial surgery.

**Figure 1:**
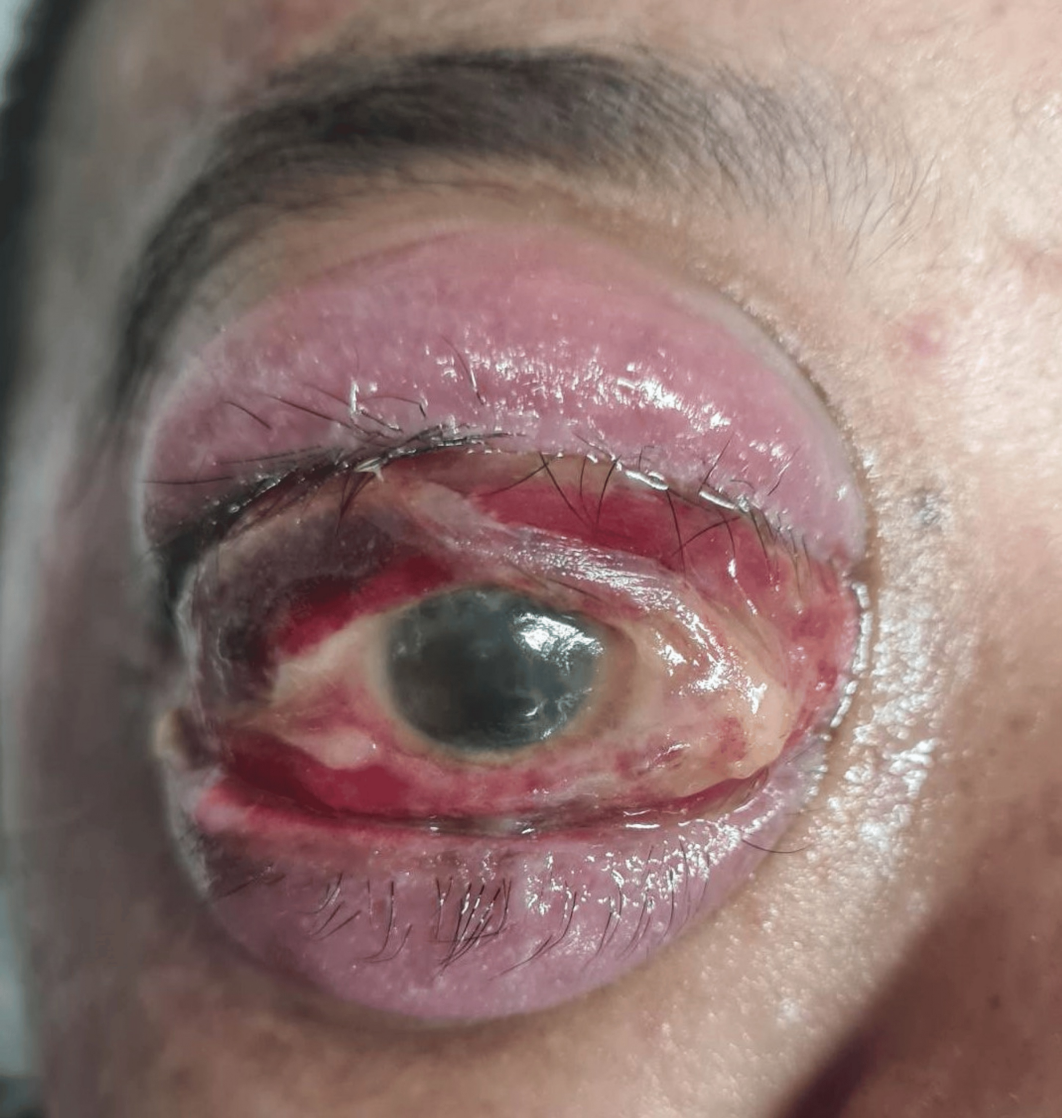
Severe proptosis, chemosis, and lagophthalmos

The patient also sustained a right periorbital haematoma but she had no visual complaints initially and was treated conservatively. Unfortunately, one week later, she developed severe right eye proptosis (Figure 1) which was pulsatile and had a bruit upon auscultation. There was profound vision loss of perception to light only, with the presence of a relative afferent pupillary defect, complete ophthalmoplegia in all gazes, extensive chemosis with cork-screw vessels, and an exceedingly high intraocular pressure (IOP) of 50 mmHg.

She was suspected clinically to have a traumatic direct carotid-cavernous fistula (CCF) with right orbital compartment syndrome. Lateral canthotomy and cantholysis was performed immediately and maximum systemic and topical IOP lowering agents were given. She was then transferred to a government hospital for further management due to financial issues.

**Figure 2:**
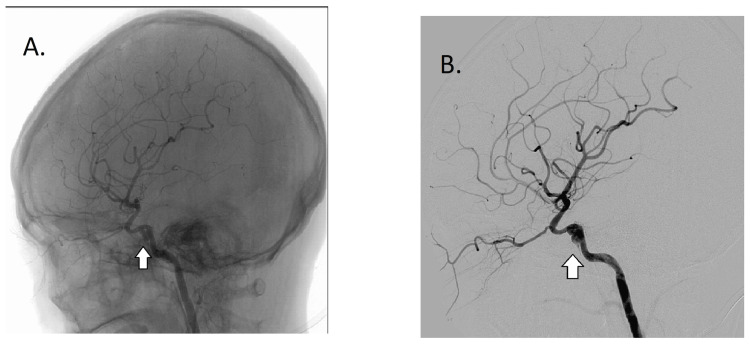
A. Cerebral digital subtraction angiography with an arrow pointing at a dissecting aneurysm of the cavernous segment of the internal carotid artery. B. Cerebral digital subtraction angiography with an arrow pointing at a dissecting aneurysm of the cavernous segment of the internal carotid artery

**Figure 3:**
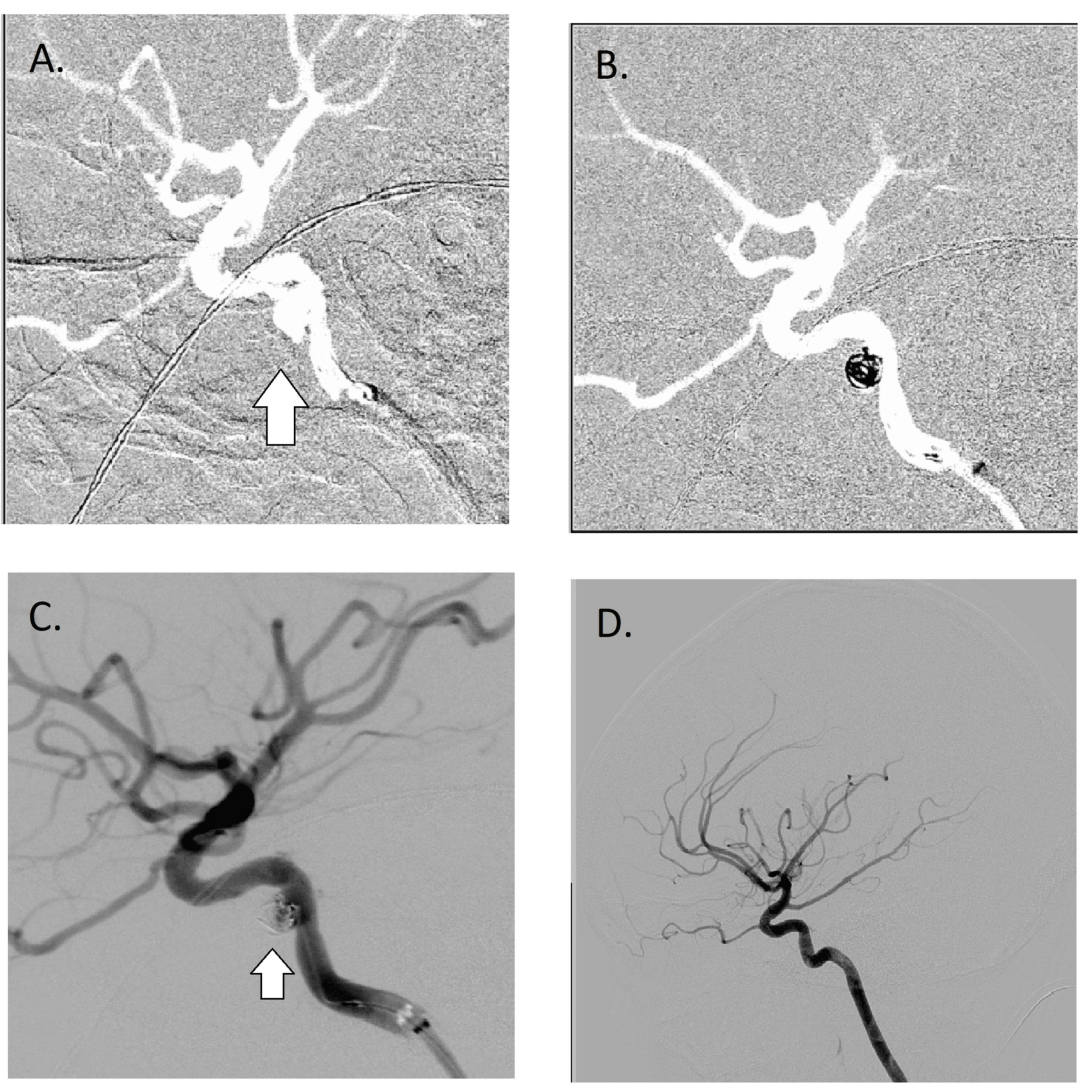
A. An arrow pointing at dissecting aneurysm of the cavernous segment of the internal carotid artery. B & C. Stenting-assisted coiling of the dissecting aneurysm of the intracavernous segment of the internal carotid artery. D. Resolution of the dissecting aneurysm of the intracavernous segment of the internal carotid artery

**Figure 4:**
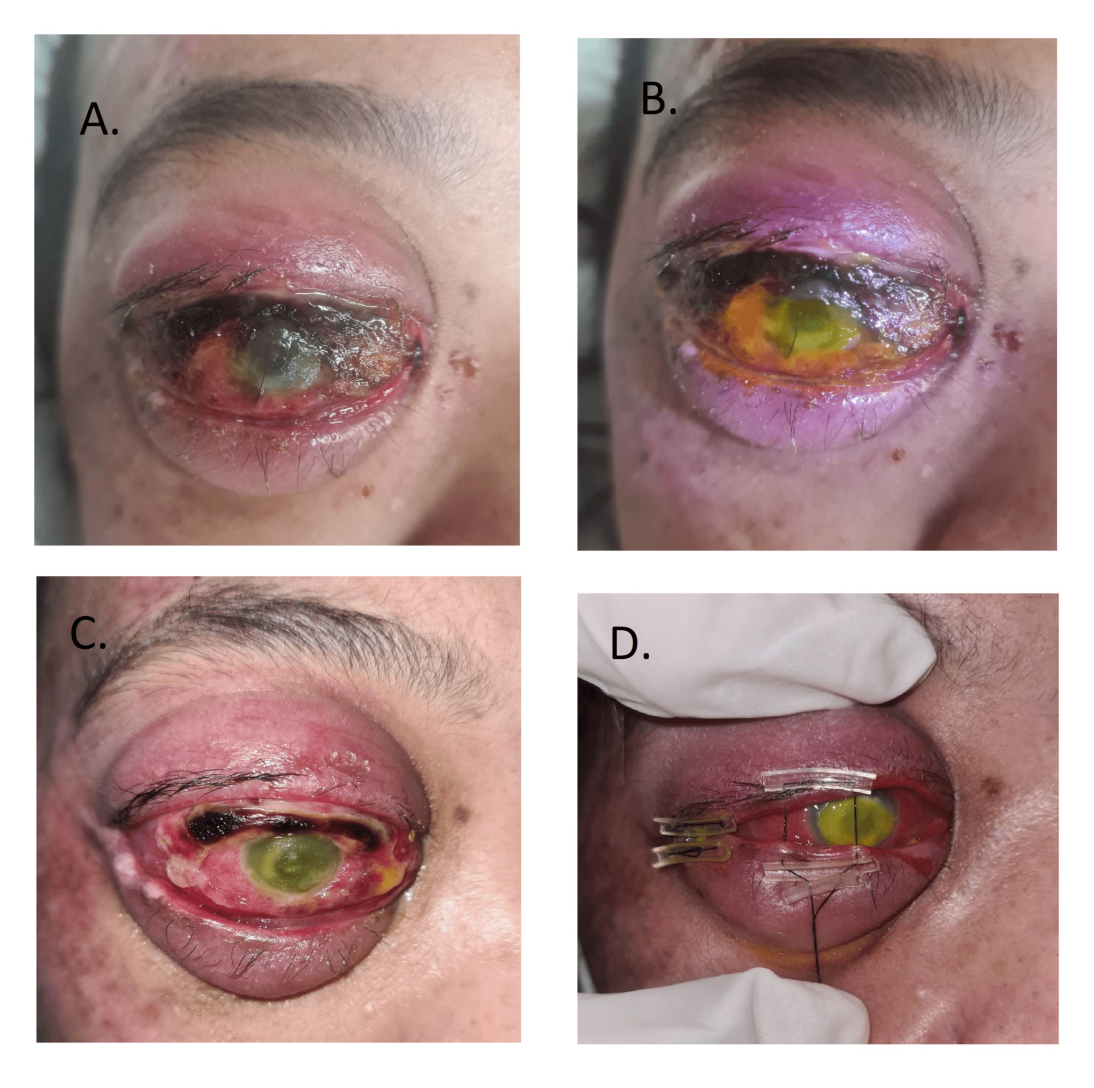
A. Severe exposure keratopathy. B. Severe fungal corneal ulcer. C. Worsening of fungal corneal ulcer. D. Temporary tarsorrhaphy

The patient was scheduled for an embolization of the fistula. However, during cerebral digital subtraction angiography, there was an incidental finding of a DA measuring 4.7 mm (height) x 4.4 mm (neck) x 7.9 mm (width) at the right ICA at the cavernous segment (Figures 2-4). A cerebral computed tomography angiography and venography showed right cavernous sinus and right superior ophthalmic vein thrombosis.

The patient was started on antiplatelet agents and later underwent successful stenting-assisted coiling of the DA of the intracavernous segment of the ICA.

Unfortunately, while awaiting definitive treatment of the DA, she developed severe exposure keratopathy (with a melting fungal corneal ulcer with a positive *Candida albicans* culture). Despite intensive ocular lubricants and antifungal eyedrops, the corneal ulcer worsened. Thus, a temporary tarsorrhaphy was done (Figure 4).

**Figure 5:**
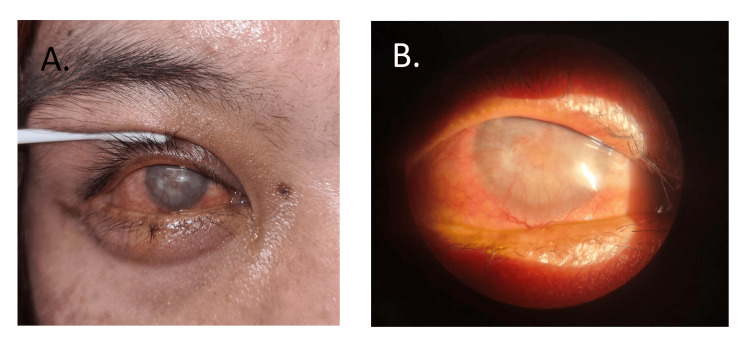
A & B. Resolution of corneal ulcer with significant corneal scar

After the successful coiling and resolution of the aneurysm, her right eye proptosis gradually resolved, and the corneal ulcer gradually healed with a significant corneal scar. The final visual acuity of her right eye was no perception of light (Figure 5). 

## Discussion

This case highlighted the concomitant rare findings of a DA of the right cavernous segment of the ICA with right cavernous sinus and right superior ophthalmic vein thrombosis following craniomaxillofacial trauma.

The pathogenesis of a DA starts with vascular wall injury with blood penetration into the vessel wall resulting in interstitial haemorrhage and dissection. Accumulated blood between the tunica intima and the media results in arterial lumen narrowing, whereas a dissection between the media and adventitia causes aneurysmal dilatation [1-3].

DAs involving the ICA following trauma can present in nonspecific ways and some literature report that it is difficult to distinguish it and thus lead to underdiagnosis [2]. Common presenting symptoms include diplopia (89%), retroorbital pain (61%), headache (19%), blurring of vision (14%), and photophobia (4%). Most cases present with partial or complete ophthalmoplegia (93%). The trigeminal nerve was also involved in 37% of patients. Other signs can include ptosis, decreased visual acuity, proptosis, and visual field defects [4]. In rare cases, they can present with orbital compartment syndrome [5]. The clinical manifestation depends on which segment of the ICA is involved. In this case, the patient presented with acute CSS a week after the trauma and initially was suspected to be due to a traumatic direct CCF. This is similar to cases reported in a study where the patients presented with symptoms of acute CSS, ophthalmoplegia, and pain [6].

The cavernous sinuses are small, paired venous structures, each measuring approximately 3 cm long, 1 cm wide, and 0.5 cm high, that lie on either side of the pituitary fossa. There are numerous important structures that run through the cavernous sinus such as the ICA and several cranial nerves, namely the oculomotor nerve (CN III), trochlear nerve (CN IV), ophthalmic branch of the trigeminal nerve (CN V1), maxillary branch of the trigeminal nerve (CN V2), and the abducent nerve (CN VI) [7]. CSS is a condition caused by any pathology or lesion present within the cavernous sinus that disrupts the function of these anatomical structures. A review was done to characterize the cause of CSS. The study reported that tumours and traumas are the most common causes of CSS while other causes include inflammation, carotid aneurysms, CCF, and infections [8,9].

Cavernous carotid aneurysms are usually treated with aneurysm obliteration with ICA preservation and can be done with coil embolization with or without stent assistance [10-12]. In our case, our patient was given antiplatelet therapy before undergoing stent-assisted coil embolization. 

Although the aetiology of CSS is diverse, the presenting symptoms are rather similar [2,4,5]. Hence, neurovascular imaging techniques play a critical role as the diagnostic gold standard in order to define the therapeutic approach. Unfortunately, for this patient, she could not afford repeated neuroimaging and hence there was a delay in the definitive treatment when the actual cause of her symptoms was only revealed during a cerebral digital subtraction angiography. As a result, the patient sustained irreversible visual loss due to the prolonged insult from the CSS.

## Conclusions

The underlying cause of CSS is diverse and unique. Hence, the definitive treatment is distinctive and individualized. The utilization of conventional neurovascular imaging is an invaluable instrument in arriving at the proper diagnosis in a timely manner. It is of paramount importance to prevent delays to the management to maximize the chance of a favourable outcome and avoid irreversible visual morbidity.
